# Supplementary material for: From sequence to enzyme mechanism using multi-label machine learning
Source: BMC Bioinformatics. 2014 May 19;15:150. doi: 10.1186/1471-2105-15-150 (PMC4229970; doi:10.1186/1471-2105-15-150)
Supplement: Additional file 2 — Java code of ml2db. Additional file ml2db_code.tar.gz contains the Java source code to run the multi-label machine learning experiments and save the results to database. The code’s Javadoc is included. [file 1471-2105-15-150-S2.zip › additional file 2/ml2db/ml2db/doc/mulan/data/LabelSet.html]

LabelSet


JavaScript is disabled on your browser.


- Overview
- Package
- Class
- Use
- Tree
- Deprecated
- Index
- Help

- Prev Class
- Next Class

- Frames
- No Frames

- All Classes

- Summary:
- Nested |
- Field |
- Constr |
- Method

- Detail:
- Field |
- Constr |
- Method


mulan.data

## Class LabelSet

- java.lang.Object
- - mulan.data.LabelSet

- All Implemented Interfaces:
  :   java.io.Serializable, java.lang.Comparable<LabelSet>

  ---

    

  ```
  public class LabelSet
  extends java.lang.Object
  implements java.io.Serializable, java.lang.Comparable<LabelSet>
  ```

  Class that handles labelsets

  Version:
  :   $Revision: 1.1 $

  Author:
  :   Grigorios Tsoumakas, Robert Friberg

  See Also:
  :   Serialized Form

- - ### Constructor Summary

    Constructors

    Constructor and Description

    `LabelSet(double[] set)`

    Initializes an object based on an array of doubles containing 0/1
  - ### Method Summary

    Methods

    | Modifier and Type | Method and Description |
    | --- | --- |
    | `int` | `compareTo(LabelSet o)` Used for sorting collections of labelsets according to size |
    | `boolean` | `equals(java.lang.Object obj)` |
    | `static LabelSet` | `fromBitString(java.lang.String bits)` Constructs a LabelSet object from a bitstring. |
    | `java.util.ArrayList<LabelSet>` | `getSubsets()` Constructs all subsets of a labelset (apart from the empty one). |
    | `int` | `hammingDifference(LabelSet other)` Calculates the Hamming Distance between the current labelset and another labelset. |
    | `int` | `hashCode()` |
    | `static LabelSet` | `intersection(LabelSet l1, LabelSet l2)` |
    | `int` | `size()` The number of set members. |
    | `java.lang.String` | `toBitString()` Constructs a bitstring from the current labelset. |
    | `boolean[]` | `toBooleanArray()` Get an array representation of this set. |
    | `double[]` | `toDoubleArray()` Get an array representation of this set. |
    | `java.lang.String` | `toString()` A comma-separated list of label names enclosed in curlies. |

    - ### Methods inherited from class java.lang.Object

      `getClass, notify, notifyAll, wait, wait, wait`

- - ### Constructor Detail


    - #### LabelSet

      ```
      public LabelSet(double[] set)
      ```

      Initializes an object based on an array of doubles containing 0/1

      Parameters:
      :   `set` -
  - ### Method Detail


    - #### toString

      ```
      public java.lang.String toString()
      ```

      A comma-separated list of label names enclosed in curlies.

      **Overrides:**
      :   `toString` in class `java.lang.Object`


    - #### hashCode

      ```
      public int hashCode()
      ```

      **Overrides:**
      :   `hashCode` in class `java.lang.Object`


    - #### equals

      ```
      public boolean equals(java.lang.Object obj)
      ```

      **Overrides:**
      :   `equals` in class `java.lang.Object`


    - #### size

      ```
      public int size()
      ```

      The number of set members. Calculated on first call and
      cached for subsequent calls.

      Returns:
      :   The number of set members


    - #### toDoubleArray

      ```
      public double[] toDoubleArray()
      ```

      Get an array representation of this set.

      Returns:
      :   a copy of the underlying array.


    - #### toBooleanArray

      ```
      public boolean[] toBooleanArray()
      ```

      Get an array representation of this set.

      Returns:
      :   a copy of the underlying array.


    - #### hammingDifference

      ```
      public int hammingDifference(LabelSet other)
      ```

      Calculates the Hamming Distance between the current labelset and another labelset.

      Parameters:
      :   `other` - the other LabelSet object.

      Returns:
      :   the Hamming Distance.


    - #### toBitString

      ```
      public java.lang.String toBitString()
      ```

      Constructs a bitstring from the current labelset.

      Returns:
      :   the bitstring.


    - #### fromBitString

      ```
      public static LabelSet fromBitString(java.lang.String bits)
                                    throws java.lang.Exception
      ```

      Constructs a LabelSet object from a bitstring.

      Parameters:
      :   `bits` - the bitstring

      Returns:
      :   the labelset.

      Throws:
      :   `java.lang.Exception`


    - #### getSubsets

      ```
      public java.util.ArrayList<LabelSet> getSubsets()
                                               throws java.lang.Exception
      ```

      Constructs all subsets of a labelset (apart from the empty one).

      Returns:
      :   an ArrayList of LabelSet objects with the subsets.

      Throws:
      :   `java.lang.Exception`


    - #### intersection

      ```
      public static LabelSet intersection(LabelSet l1,
                          LabelSet l2)
      ```

      Parameters:
      :   `l1` - a labelset
      :   `l2` - another labelset

      Returns:
      :   their interesection


    - #### compareTo

      ```
      public int compareTo(LabelSet o)
      ```

      Used for sorting collections of labelsets according to size

      **Specified by:**
      :   `compareTo` in interface `java.lang.Comparable<LabelSet>`


- Overview
- Package
- Class
- Use
- Tree
- Deprecated
- Index
- Help

- Prev Class
- Next Class

- Frames
- No Frames

- All Classes

- Summary:
- Nested |
- Field |
- Constr |
- Method

- Detail:
- Field |
- Constr |
- Method
